# Investigating Various Permutations of Copper Iodide/FeCu Tandem Materials as Electrodes for Dye-Sensitized Solar Cells with a Natural Dye

**DOI:** 10.3390/nano10040784

**Published:** 2020-04-19

**Authors:** Abdul Hai Alami, Mohammed Faraj, Kamilia Aokal, Abdullah Abu Hawili, Muhammad Tawalbeh, Di Zhang

**Affiliations:** 1 Sustainable and Renewable Energy Engineering Department, University of Sharjah, Sharjah 27272, UAE; mtawalbeh@sharjah.ac.ae (M.T.); zdi@sharjah.ac.ae (D.Z.); 2 Center for Advanced Materials Research, Research Institute of Science and Engineering, University of Sharjah, Sharjah 27272, UAE; mfaraj@sharjah.ac.ae (M.F.); k_aokal@outlook.com (K.A.); 3 Sharjah Research Academy, Sharjah 27272, UAE; a.abouhewelle@sra.ae

**Keywords:** copper iodine, FeCu alloys, ball milling, dye-sensitized solar cells, natural sensitizers, *Calotropis gigantea*

## Abstract

This work presents the synthesis and deposition of CuI and FeCu materials on copper substrates for dye-sensitized solar cell applications. FeCu is a metastable alloy of iron and copper powders and possesses good optical and intrinsic magnetic properties. Coupled with copper iodide as tandem layers, the deposition of these two materials was permutated over a pure copper substrate, characterized and then tested within a solar cell. The cell was sensitized with a natural dye extracted from a local desert plant (*Calotropis gigantea*) and operated with an iodine/triiodide electrolyte. The results show that the best layer arrangement was Cu/FeCu/CuI, which gave an efficiency of around 0.763% (compared to 0.196% from reported cells in the literature using a natural sensitizer).

## 1. Introduction

Dye Sensitized Solar Cells (DSSC) are the most investigated third-generation solar cell technologies due to their low cost, ease of fabrication, and relatively high efficiencies (about 10%) [1,2]. They were introduced by O’Regan and Gratzel who employed nanocrystalline mesoporous TiO_2_ films and reported a very high conversion efficiency of around 7.1–7.9% and 12% in simulated solar light and diffuse daylight, respectively [3].

Extensive research has been conducted to enhance the performance of DSSC by modifying the physicochemical properties of the main components of the cells. Most of this research focused on exploring new dyes, increasing the surface area of the mesoporous layer and improving the working electrode materials and configurations [4]. Lim et al. reported that enhancing DSSC by the use of an ethylene glycol-based polymer gel electrolyte with ZrO_2_ nanofillers resulted in a 5.6% power conversion efficiency [5]. Iglesias et al. showed, in their study, that the use of carbon nanohorns and their resultant dye conjugates led to an increase in the DSSC efficiency from 4.07% to 6.24% [6]. Another study on the modification of the DSSC by Li et al. showed the use of ZnO hollow microspheres coated by TiO_2_ in Quantum Dots DSSC to enhance the light harvesting efficiency of the cell, resulting in a power conversion efficiency of 3.16% compared to 1.54% in the absence of the TiO_2_ coating [7]. Moreover, in their attempt at commercializing DSSC, Cheng et al. reported the development of a quasi-solid DSSC with a long-term stability with a conversion efficiency of 8.63% [8]. In addition, due to intensive research, the efficiency of the DSSC reached a record value of 14.30% in 2015 [9]. Moreover, metal oxides are among the materials that are commonly investigated for their role in enhancing the efficiencies of DSSCs [10,11]. They are used for enhancing the mesoporous layers [12], bandgap engineering [13] and in counter electrodes enhancements [14]. For example, Sharma et al. reported that using ZnO nanolayers in DSSCs resulted in an efficiency of 0.49% [15].

Today, more research is directed toward examining new materials as counter electrodes [16]. Platinum (Pt), despite its high cost, is the most commonly used material as counter electrodes. Several lower-cost materials were considered in the literature to substitute Pt, such as tungsten, selenide, nickel, aluminum, ruthenium, copper, zinc, conducting polymers and carbonaceous materials [17,18]. For example, Vijaya et al. reported that MoS_2_ nanosheets-based counter electrodes coupled with reduced graphene oxide for DSSC exhibited a fill factor of 0.42, a power conversion efficiency of 8.1% compared with 0.47 fill factor, and a power conversion efficiency of 6.8% obtained by using Pt-counter electrodes [19]. Another attempt at making Pt-free counter electrodes by Huang et al. showed a power conversion efficiency of 9.21% utilizing a nano yolk-shell structure of nitrogen-doped CoS_2_ [20]. A study by Kim et al. reported that integrating Tellurium-doped mesoporous carbon nanomaterials as a transparent counter electrode for bifacial DSSC resulted in 9.43% and 8.06% power conversion efficiencies under front and rear-side irradiation, respectively [21]. Moreover, Nan et al. showed an economic replacement of Pt counter electrodes using NiCo_2_S_4_/reduced graphene oxide with a 6.01% efficiency [22].

Copper, in particular, has several properties that deem it as a favorable candidate for various electrical and thermal applications. These include its high thermal conductivity, ease of fabrication, availability, relatively low cost, durability, high electrical conductivity and resistance for corrosion [14]. Copper is typically combined with other materials to better serve certain applications. For instance, cupric oxide (CuO) and cuprous oxide (Cu_2_O) have band gaps of 1.21–2.1 eV and 2.2–2.9 eV, respectively, which made them very attractive as visible light absorption materials [23,24]. Zhuang et al.’s study highlighted that electron transport could be enhanced by introducing copper into the ZnO layer in photoelectrodes resulting in a 1.6 times enhancement of the power conversion efficiency [25]. DSSC with Pt-free counter electrodes of Cu_2_ZnSnS_x_Se_4-x_ nanosheets on FTO substrate was reported on by Mohammadnezhad et al. to have a 5.73% power conversion efficiency compared to 5.78% using the Pt-based CE [26]. Moreover, cuprous iodide (CuI) is also used as a hole transporter in DSSC [27]. CuI has very high hole mobility and transparency, it is chemically stable, easy to deposit, and has a low production cost [28,29]. It is worth mentioning that the most commonly employed electrolyte is iodide/triiodide [30,31].

The Fe-Cu bimetallic alloy has been integrated into several applications due to its notable mechanical electrical and optical properties [32,33]. It was integrated in a previous work by Alami et al. as a mesoporous layer replacing the TiO_2_ layer in DSSC to a good degree of success [34]. 

The photosensitizers in DSSC are classified principally as three main types; (i) metallic complexes—specifically ruthenium(II)—(ii) zinc porphyrin derivatives, and (iii) organic dyes [2,35]. In the literature, several natural dyes were utilized to prepare DSSC, such as spinach, rosella, red beet roots, red cabbage, red turnip, rhoeo spathacea stream, curcumin, red perilla, sesbania grandiflora scarlet, achiote seed, blue pea, and pomegranate [36]. The dye extracted from Calotropis is a natural dye; hence, it is expected to contain a combination of pigments. This combination is anticipated to extend the absorption range beyond that of a single pigment, which is believed to improve the DSSC efficiency [37].

In this work, the synthesis and deposition of tandem layers of FeCu and CuI on a copper substrate was investigated for dye-sensitized solar cells application. The available permutations of materials arrangement were executed (i.e., Cu/CuI, Cu/FeCu/CuI and Cu/CuI/FeCu), and their effect on the solar cell performance (mainly the efficiency) is reported. The cells were sensitized via a natural dye and thus, the cells were also compared to the performance of their counterparts reported in the reviewed literature. The pertinent microstructural and optical characterizations are also reported.

## 2. Materials and Methods 

The following paragraphs detail the synthesis and deposition of the materials on the copper substrates. The solar cell construction and testing are also specified. High-quality copper sheets 0.2 mm in thickness and measuring 1 × 10 cm were used as substrates for the deposition of CuI and FeCu thin films. The sheets were initially sonicated in a solution containing a 1:1 volume ratio of ethanol to acetone for one hour at 55–60 ˚C to remove any organic contaminants.

### 2.1. Synthesis of the FeCu Material

Binary metastable Fe-Cu powder was synthesized via dry-ball milling with a 1:1 mass ratio of pure iron and copper powders (used as received from Sigma-Aldrich). The mechanical alloying took place in the Fritsch (FRITSCH GmbH Idar-Oberstein, Germany) premium line Pulverisette 7 planetary ball mill at a 600 rpm milling speed for ten hours milling time. The ball to powder ratio was fixed at 6:1.

**Figure 1:**
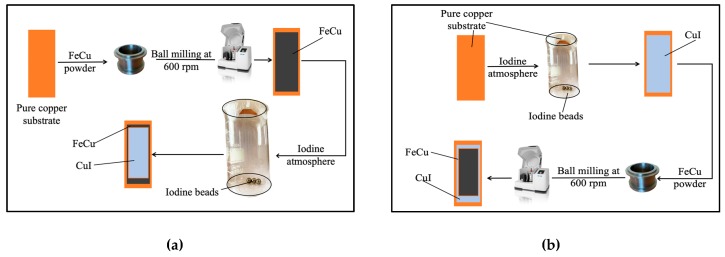
(**a**) FeCu deposition on pure Cu substrate followed by CuI growth on the resulting Cu/FeCu collar and (**b**) CuI growth on Cu substrate followed by ball-less milling of a Cu/CuI collar to deposit FeCu on CuI.

The ball mill machine was also used in order to apply a uniform layer of coating on the used electrodes by virtue of the kinetic energy and the centrifugal motion of the milling and without using balls. For this part of the experiment, three grams of previously prepared FeCu powder was placed in a crucible along with the thin copper substrate fixed on the walls (as a collar). The milling speed was fixed at 600 rpm, maintained for two consecutive hours until a layer of FeCu was obtained on the Cu/CuI collar, as depicted in Figure 1a.

### 2.2. Synthesis of Copper Iodide (CuI)

The Cu sheets were dried by N_2_ gas and transferred into the route of CuI deposition. This involved the growth of an intrinsic layer of CuI on the surface of the copper sheets and took place in a beaker sealed with parafilm that contained 6 mmol pure Iodine beads. The beads decompose at room temperature, generating corrosive I_2_ fumes that reduce copper (II) ions to form copper (I) iodide on the surface of the substrate and thus, generate an adherent gray-colored CuI film. Figure 1b shows the process of growing the CuI film and the subsequent milling of FeCu powder to add the FeCu on top of CuI.

Following a two-step process, iodide is oxidized by Cu^2+^ ions to form intermediate CuI_2_.

(1) $$Cu^{2+}+2I^{-}\to Cu^{+}+I_{2}$$

CuI_2_ is unstable due to the strong reducing properties of iodide, which reduces Cu^2+^ to Cu^+^.

(2) $$CuI_{2}\to CuI+\frac{1}{2}I_{2}$$

CuI has three known solid phases. At room temperature and below 369 ˚C, it arranges with a face-centered cubic (FCC) crystal structure (known as γ-CuI). Between 369 ˚C and 407 ˚C, it forms a hexagonal close-packed (HCP) structure (β-CuI). Above 407 ˚C, it forms a disordered face-centered cubic (FCC) structure (α-CuI).

Having four lone pairs, iodine potentially coordinates to four surrounding atoms. For an enhanced catalytic response, however, reducing the coordination number to two copper atoms allows the interaction between copper centers and externally supplied ligands and sensitizers. Since the coordination of copper (I) to four ligands is energetically favorable, this augments the chances of dye molecules intercalation and potential interaction with incident solar radiation. Moreover, according to Yang et al., CuI behaves more like a p-type semiconductor, contrarily to TiO_2_ [38]. 

### 2.3. Microstructural Characterization of Material

The microstructure of the produced electrodes was examined by scanning electron microscopy (SEM), both regularly, using a VEGA3 TESCAN (Tescan Analytics, Actipôle St Charles, Fuveau, France) electron microscope operating at 30 kV acceleration voltage used to investigate the topography of the produced material, and with an Apreo C field emission (FE-SEM) from Thermoscientific (Thermo Fisher Scientific, Brno, Czech Republic) operating at low vacuum and 15 kV with an electrostatic lens. The TESCAN SEM has a coupled provision for energy dispersive x-ray spectroscopy (EDS) and was also used to take cross-sectional SEM images of the electrodes. X-ray diffraction (XRD) measurements were also taken for the electrodes on a Bruker (Bruker GmbH, Wien Austria) D8 Advance Da Vinci multipurpose X-ray diffractometer, with Cu Kα radiation operating at λ = 1.5406Å. Raman spectroscopy was performed at room temperature in backscattering configuration with a Renishaw inVia Raman microscope (Renishaw, gloucestershire UK) via its visible 514 nm laser at a resolution of 1cm^−1^.

Spectral absorbance in the Vis-NIR range (375–815 nm) of the films was carried out via a Maya 2000-Pro high-resolution spectrometer (Ocean Optics Inc.,Photonic Solutions, Edinburgh, UK) with a 10-µm entrance slit. The spectrometer has a resolution of 0.2 nm and 300 lines per mm diffraction gratings. The signals are transported via a fiber optic cable that is 2-m long, with a 200-µm-core diameter. The integrating sphere (OceanOptics ISP-REF, with a sample aperture of 1.016 cm) has a built-in tungsten halogen light source (Ocean Optics LS-1-LL) and was used to measure the films under the effects of reflectance. A reference surface of a pure copper substrate was used to store baseline reflectance (100%) spectra to facilitate comparison between the various samples. 

### 2.4. Dye Extraction and Characterization

The natural sensitizer for this project was extracted from the *Calotropis gigantea* plant that grows naturally in the United Arab Emirates. *Calotropis gigantea* leaves were washed thoroughly with water and liquid soap to remove any surface impurities and were left to dry in air. The leaves were then cut up into smaller pieces with an average surface area equal to 0.25 cm^2^, avoiding the midrib and branched out veins. The leaves were then sonicated for an hour in a mixture containing a 3:1 mass ratio of the leaves to ethanol and were subsequently filtered through a PTFE syringe filter of 0.45 µm pores for electrode adsorption. 

The dye characterization included a Fourier Transform Infrared Spectrum (FTIR) analysis done using ATR-FTIR spectra, recorded on a Burker Platinum ATR Tensor II FT-IR (Bruker GmbH, Wien Austria) spectrophotometer within the wave band of 4000~500 cm^−1^. 

### 2.5. Solar Cell Assembly 

**Figure 2:**
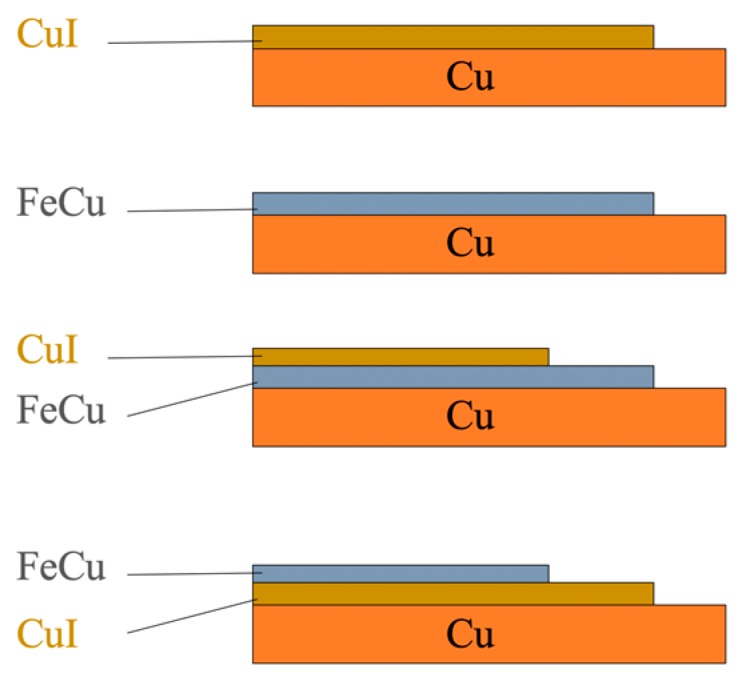
Deposition order of the FeCu and CuI materials on pure copper substrate (top to bottom: Cu/CuI, Cu/FeCu, Cu/FeCu/CuI and Cu/CuI/FeCu).

To study the effect of this tandem deposition on the performance of the solar cell device that is intended for this study, four electrode permutations were produced, as seen in Figure 2. The variation of the spectral and microstructural properties of these layers was examined to arrive at the most desirable properties for the best device performance.

The construction and testing of the cells were arranged to follow an inverted stratification, where the negative electrode is one of the combinations (highlighted in Figure 2) with an added layer of titanium dioxide (TiO_2_), and the positive terminal is the Pt photoanode.

A standard Ti-nanoxide T/SP paste from Solaronix (Solaronix, Aubonne, Switzerland) was drop-cast onto the aforementioned copper substrates occupying an active area of ~ 0.25 cm^2^. The electrodes were heated up to 550 ˚C until there was a noticeable color change, a process that took around 30 min. The electrodes were then placed in the natural dye overnight for dye adsorption.

The positive electrode comprised of a ready-made 0.2 cm thick platinum (Pt)-coated (screen printed) Platisol T/SP precursor onto fluorine-doped tin oxide (FTO) glass measuring 2 × 2 cm from solaronix. Iodolyte AN-50 was used as the electrolyte, also from Solaronix.

**Figure 3:**
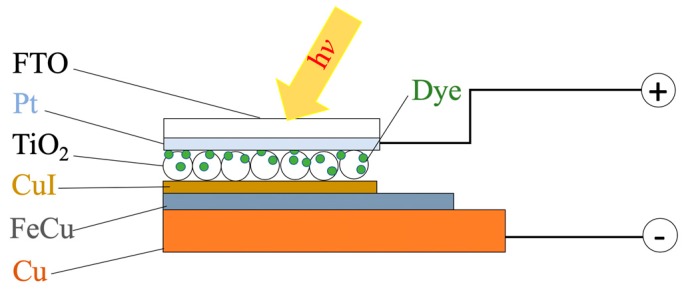
The assembled device (solar cell), featuring the Cu/FeCu/CuI electrode.

A sample of the assembled device is shown in Figure 3, featuring the Cu/FeCu/CuI electrode. 

### 2.6. Solar Cell Testing and Characterization

The current density-voltage (J-V) measurements of the DSSC were carried out on a Keithley 2400 SourceMeter under an ABET SunLite solar simulator at AM1.5G and 1000 W m^−2^ irradiance at room temperature.

## 3. Results

The following paragraphs present the main results obtained from the deposition and characterization of the dye and electrodes materials, as well as the tests conducted to characterize the produced solar cells.

### 3.1. Microstructural characterization 

#### 3.1.1. Scanning Electron Microscopy (SEM)

**Figure 4:**
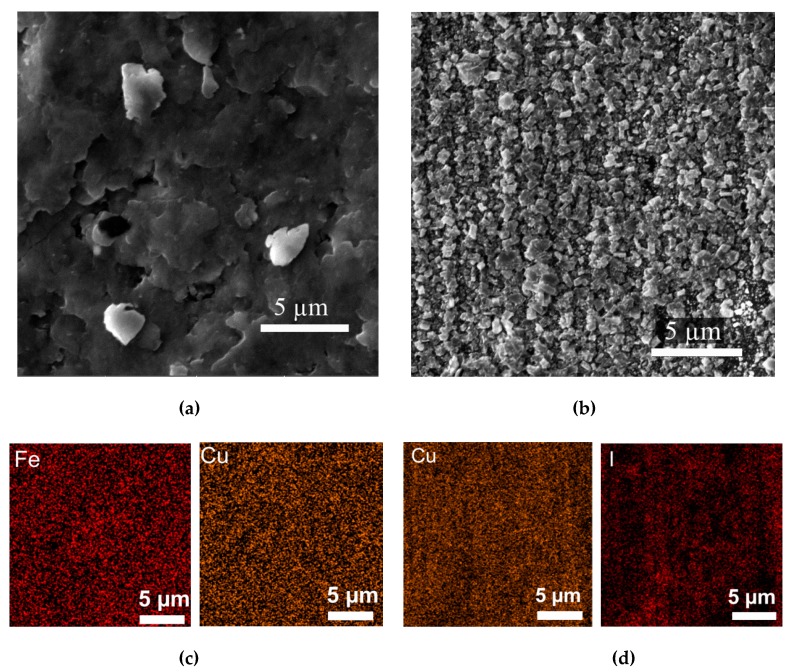
(**a**) FeCu deposition on the copper substrate, (**b**) CuI growth on Cu, (**c**) EDS mapping of the FeCu distribution and (**d**) EDS mapping of the CuI distribution.

The material deposition was first verified via SEM measurements. The FeCu and CuI deposition on the Cu substrate is shown in Figure 4a,b, respectively. The former depicts the homogenous FeCu coverage of the Cu substrate due to the high energy involved in depositing the FeCu powder on the Cu surface, while Figure 4b shows the growth of CuI on the copper substrate, with a homogenous granular coverage of the substrate too. The EDS measurements shown in Figure 4c,d confirm the homogeneity, with 75.2% wt. copper and 24.8% wt. I for the CuI, while ~70% wt. Cu and 30% wt. Fe (due to interference from the copper substrate) is evident from the FeCu deposition EDS.

**Figure 5:**
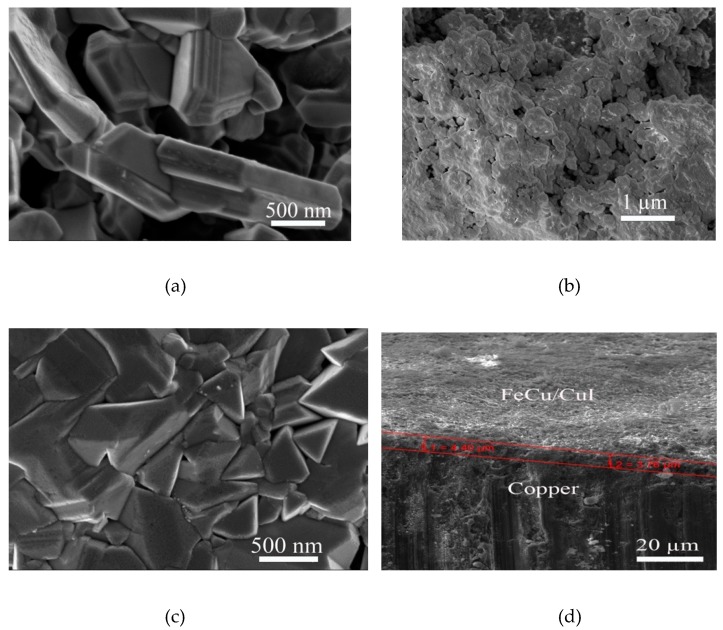
(**a**) Field emission scanning electron microscopy (FE-SEM) micrographs of (**a**) copper iodine growth over FeCu deposited on copper substrate, (**b**) FeCu ball-milled over CuI deposited on the cipper substrate, (**c**) CuI growth on copper substrate, showing monolithic CuI growth and (**d**) cross sectional SEM of FeCu/CuI on copper substrate.

Field emission SEM (FE-SEM) was also used to highlight the deposition permutation of (i) FeCu/CuI and (ii) CuI/FeCu on the copper substrate, as shown in Figure 5a,b, respectively. It is also used to further examine the homogeneity of copper iodine formation on the copper substrate, as seen in Figure 5c.

The unimpeded CuI growth over the copper substrate is seen to commence growth as monolithic columns, then break down into triangular grains (see the contrast between Figure 4b and Figure 5c). On the other hand, the intrinsic high-energy nature of FeCu deposition shows surfaces that are more homogenous and exhibit a suppressed microstructure, mainly due to the physical erosion that accompanies the centrifugation that takes place in the milling apparatus. 

The effect of the microstructure on the performance of the produced devices was further investigated spectrally, as the physical properties affect the direct bandgap of the materials, and hence, the interaction with incident electromagnetic waves during solar cell testing. The tandem material deposition (e.g., FeCu/CuI on copper) is stable and compact, again due to the energy in the milling machine that produces the adherent powder. The thickness of the material was measured using a cross-sectional SEM measurement shown in Figure 5d, where the layer has a thickness of 3.76–4.40 µm.

#### 3.1.2. Raman Spectroscopy and X-ray Diffraction

**Figure 6:**
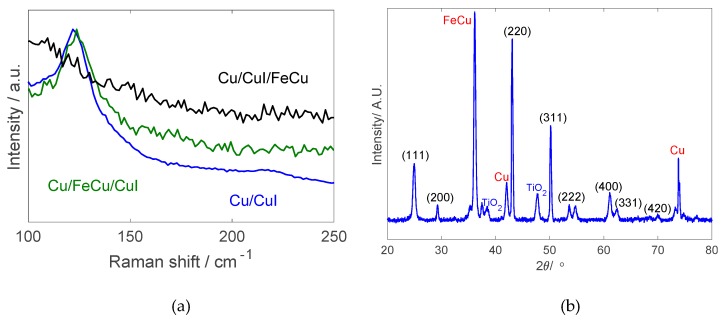
(**a**) Raman spectroscopy for all electrode deposition permutations and (**b**) X-ray diffraction patterns featuring the Cu/CuI/Cu electrode (crystallographic planes are shown for CuI).

Raman spectroscopy was performed on all produced electrodes with CuI films and the spectra are shown in Figure 6a. The optical transverse mode of CuI bonding vibration is visible at a peak of 120 cm^−1^ [39], after which the curves show similar trends as that of FeCu thin film as the top layer. Specimens with FeCu as the top layer do not show that particular peak, with all single points taken exhibiting the same behavior due to the strong scattering effect of the FeCu layer which has the ability to reduce the penetration depth of the 514 nm laser. Therefore, the existence of CuI on this electrode is more clearly depicted in the XRD spectra of Figure 6b with an added TiO_2_ layer. 

The XRD patterns of the Cu/CuI/FeCu electrode shown Figure 6b indicate the existence of all the expected compounds. The measured electrodes, in this case, with the added TiO_2_ layer, show strong signatures of FeCu, Cu, CuI as well as TiO_2_, where the CuI peaks are indicated with their corresponding crystallographic plane number. The XRD patterns are identical for all permutations of the electrodes produced, and hence, Figure 6b is representative of all cases, as the penetration of the X-rays covers the thin tandem materials and into the copper substrate.

### 3.2. Spectral Absorptivity of Electrodes and FTIR Analysis of the Natural Dye Sensitizer

**Figure 7:**
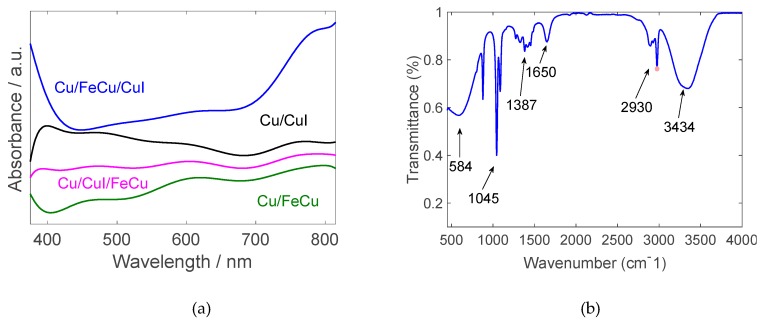
(**a**) Spectral absorptivity of produced electrodes and (**b**) FTIR spectra of the natural Calotropis dye.

The absorbance of all produced electrodes is shown in Figure 7a. Two broad peaks are observed for FeCu-coated copper, one at 475 nm and another at 600 nm. The latter could be attributed to the formation of charge transfer bands, owing to FeCu accepting electrons from the substrate. There seems to be a red shift in those very peaks as the effect is more pronounced in the Cu/FeCu/CuI structure. It can also be noticed that CuI absorbance peaks occur at shorter wavelengths preceding 410 nm while the absorbance decreases with the wavelength [40].

The FTIR spectra shown in Figure 7b fall within the wave band of 4000~500 cm^−1^. The figure identifies the functional groups present in the examined dye samples. The obtained spectrum is in agreement with that found in the literature for chlorophyll extracts. Natural dyes are known to be effective sensitizers for the TiO_2_ mesoporous structure and Chlorophyll has a bandgap of around 1.88 eV, which is excellent to enhance the VIS-NIR absorptivity of the cell [41].

### 3.3. Solar-Cell Testing Results

The intended application for the produced tandem material is its incorporation into solar cells sensitized by an organic dye extracted from a natural non-food plant that is abundant in the United Arab Emirates (the *Calotropis gigantea*). Some of the measured electrical characteristics of each electrode combination are shown in Table 1.

**Figure 8:**
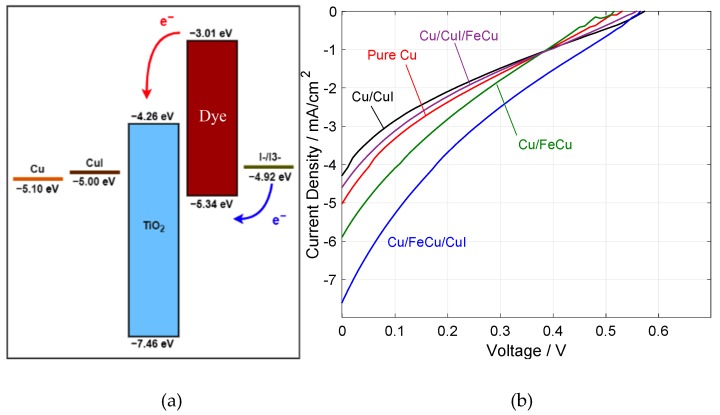
(**a**) Band diagram of various DSSC components and (**b**) J-V characteristic curves for all electrode combinations.

Considering the high cost of transparent conducting oxides (TCOs), it is very attractive to develop low-cost Cu electrodes for DSSC. However, the stability of Cu is a serious concern as it is well known to diffuse in silicon in first-generation solar cells, creating active recombination centers [43]. Furthermore, the high work function (WF) of Cu (5.10 eV, see Figure 8a) compared to the conduction band edge of TiO_2_ suggests the insertion of interface materials for efficient electron extraction. In these regards, we investigated DSSC with different Cu-based negative electrode structures, with the cell J-V characteristics shown in Figure 8b, and the extracted cell parameters are presented in Table 2.

As a reference, DSSC using pure Cu negative electrode showed a moderate performance, with a 0.497% power conversion efficiency (PCE). Notably, its low fill factor (FF) and short-circuit current density (J_SC_) (18% and 5.00 mA/cm^−2^, respectively) are strong indications of the considerable extraction barrier between Cu and TiO_2_, as shown in the energy diagram (Figure 8a). 

With a slightly lower WF of 5.00 eV compared to that of Cu, we expected CuI to mitigate the electron extraction losses by providing a more gradual energy step. Indeed, DSSC with all tested variations of CuI (Cu/CuI, Cu/CuI/FeCu and Cu/FeCu/CuI) unanimously exhibited higher open-circuit voltages (V_OC_) compared to the reference (0.570 V, 0.560 V, 0.570 V, respectively, vs. 0.530 V). However, Cu-negative electrodes modified with direct CuI deposition (Cu/CuI and Cu/CuI/FeCu) resulted in inferior cell PCEs mainly from lower J_SC_. We believe that this could be attributed to the inability of CuI to prevent Cu diffusion and the creation of active recombination sites; on the contrary, the diffusion may be facilitated by CuI given its active nature and similar composition to Cu, leading to a deteriorated performance. 

In contrast, the results of FeCu-incorporated DSSC seem to suggest a complementary role of the FeCu alloy. While V_OC_ was not improved directly in the Cu/FeCu cell (suggesting that it is not more energetically favorable compared to CuI), cell PCE was increased considerably compared to the reference (from 0.497% to 0.572%). This improvement is likely due to the stabilizing effect of FeCu deposited on Cu against Cu diffusion, which was absent in the structure of Cu/CuI/FeCu where CuI prevented FeCu from stabilizing the Cu electrode (indicated by its similarly low performance compared to Cu/CuI). Finally, the most efficient electrode design was found in the Cu/FeCu/CuI cell, where the full potential of FeCu and CuI (Cu diffusion stabilizing and electron transport, respectively) was fully achieved, giving rise to a high J_SC_ of 7.59 mA/cm^−2^, V_OC_ of 0.570 V and PCE of 0.763%. While synthetic Ru-based sensitizers are able to achieve a high PCE in DSSCs, they suffers from major drawbacks, such as a very high cost due to the rarity of the metal, complex synthesis and purification process and low stability [9,10,11]. 

In light of these limitations, natural sensitizers provide a promising alternative for realizing cost-effective DSSCs due to the abundance of natural dyes and the usually simple preparation procedures. Nevertheless, the development of DSSCs based on natural sensitizers (such as the Calotropis dye proposed in this work) is still in early stages as the most efficient device only showed PCEs of 0.278%–0.50% [31,35], which are within the range of the performance reported in this work. We would also like to point out that the obtained PCEs reported here are in combination with the use of copper-based counter electrodes which traditionally offer much lower costs at the expense of a lower performance compared to Pt. We believe that the strategy reported here is applicable to more efficient DSSC architectures, which would constitute a future study.

## 4. Conclusions

This work presents the synthesis and characterization of various materials applied to a copper substrate. As a cheap and available material, copper poses some challenges when used for solar cell applications. Here, we showcased two materials deposited on a copper substrate, namely copper iodine and FeCu. The former was chosen due to its compatibility with the iodine/triiodide electrolyte often used in dye-sensitized solar cells and also because it can be intrinsically grown on the copper material. FeCu, on the other hand, is an alloy that has distinct optical and structural properties that are worth investigating. The two materials were added in tandem for the solar cell application, and the synthesis parameters as well as characterization show the facile and rapid implementation, which would positively affect time and cost required for cell manufacture, with amenable properties both optically (e.g., bandgap) and structurally. The produced cells were sensitized with a natural dye extracted from the leaves of a locally abundant plant (*Calotropis gigantea*), that is, cannot be used as a food item for neither people nor livestock due to the irritating secretions from the stems. The efficiencies of the solar cells show the positive effect of adding FeCu on top of the copper substrate while adding the CuI successively over the FeCu. The bandgap match of these two substrates allowed a better spectral interaction with incident radiation during testing, while CuI had a better compatibility with the electrolyte. The reported efficiency of 0.763% for the Cu/FeCu/CuI arrangement is higher than the values reported in the literature for similar chlorophyll II-sensitized solar cells (~0.19%). This is a promising development, especially since the price of such cells with a copper electrode is low compared with the more expensive platinized materials and would enhance the figure of merit ($/W) once further development on the materials and structure is attempted in future work.

## Figures and Tables

**Table 1:** Electronic and energy quantities for the produced electrodes.

Material	Electron Affinity (EA)/e.V	Work Function (WF)/e.V	Ionization Energy (IE)/e.V	Energy Bandgap/e.V
Cu	1.235 [1]	4.53–5.10 [3]	7.7264 [5]	-
CuI	2.1 eV [2]	4.9–5.0 [4]		2.79 exp [42]
FeCu	NA	NA	NA	1.92 exp [34]

**Table 2:** Performance characteristics of DSSC constructed using all negative electrode structures.

Negative Electrode Structure	*J_SC_* (mAcm^−2^)	*V_OC_* (V)	FF (%)	PCE (%)
Pure Cu	5.00	0.530	0.187	0.497
Cu/CuI	4.27	0.570	0.184	0.448
Cu/FeCu	5.88	0.520	0.187	0.572
Cu/CuI/FeCu	4.58	0.560	0.182	0.467
Cu/FeCu/CuI	7.59	0.570	0.177	0.763
Calotropis/Pt (reported) [31]	0.518	0.533	0.71	0.196
